# Fermented Purslane (*Portulaca oleracea* L.) Supplementation Enhances Growth and Immune Function Parallel to the Regulation of Gut Microbial Butyrate Production in Weaned Piglets

**DOI:** 10.3390/microorganisms12071403

**Published:** 2024-07-11

**Authors:** Lei Xu, Ge Gao, Zian Zhou, Zixi Wei, Wenjuan Sun, Yanpin Li, Xianren Jiang, Jingang Gu, Xilong Li, Yu Pi

**Affiliations:** 1 Key Laboratory of Feed Biotechnology of Ministry of Agriculture and Rural Affairs, Institute of Feed Research, Chinese Academy of Agricultural Sciences, Beijing 100081, China; xlei0611@163.com (L.X.); 82101192355@caas.cn (G.G.); 13126830289@163.com (Z.W.); sunwenjuan@caas.cn (W.S.); liyanpin@caas.cn (Y.L.); jianxianren@caas.cn (X.J.); 2 Institute of Agricultural Resources and Regional Planning, Chinese Academy of Agricultural Sciences, Beijing 100081, China; 17356536652@163.com (Z.Z.); gujingang@caas.cn (J.G.)

**Keywords:** fermented purslane, immunoregulation, microbial butyrate synthesis, diarrhea, piglet

## Abstract

Weaning is a challenging period for piglets, characterized by stress-related growth checks, compromised immunity, and gut dysbiosis. Purslane (*Portulaca oleracea* L.), known for its rich content of antioxidants, has potential as a functional feed ingredient. This study investigates the effects of feeding fermented purslane (FP) on the growth performance, immune function, intestinal microbiota, and metabolic profiles of weaned piglets. Forty-eight weaned piglets were randomly divided into two groups, with eight pens in each group and three pigs in each pen: a control diet (CON group) and a diet supplemented with 0.20% FP (FP group). The experiment lasted 28 days. The results show that FP supplementation did not affect the average daily feed intake (ADFI) but significantly increased the average daily gain (ADG) during the initial 14 days post-weaning. FP supplementation decreased diarrhea occurrence, with a pronounced reduction from days 10 to 13 (*p* < 0.05). Immunologically, the FP group had a trend towards reduced serum IgA levels on day 14 (*p* < 0.10). Importantly, the serum concentrations of the pro-inflammatory cytokine IL-6 were significantly reduced on both days 14 and 28 post-weaning. The antioxidative analysis showed increased serum superoxide dismutase (SOD) and decreased catalase (CAT) activities on day 14 (*p* < 0.05). In addition, FP supplementation significantly decreased serum diamine oxidase (DAO) activity and D-lactate levels by day 28, indicating a potential improvement in gut integrity. Fecal microbiota assessment demonstrated a distinctive clustering of microbial communities between the FP and CON groups, with an increase in the abundance of *Clostridium_sensu*_stricto_1, *Tyzzerella*, and Prevotellaceae_NK3B31_group and a decrease in *Lactobacillus*, *Bacillus*, and *Subdoligranulum* in the FP group (*p* < 0.05). Functional predictions suggested that the relative abundance of microbial butyrate synthesis enzymes (EC 2.7.2.7 and EC 2.3.1.19) was significantly enhanced by FP treatment. This modulation was further corroborated by elevated fecal butyrate levels (*p* < 0.05). In summary, dietary supplementation with FP promotes early-growth performance and has beneficial effects on immune function and intestinal health in weaned piglets. The enhancements may be attributed to distinct microbiota compositional changes and targeted modulation of microbial butyrate metabolism, which are crucial for piglet post-weaning adaptation and overall health.

## 1. Introduction

The transition from sow milk to solid feed during the weaning phase is one of the most stressful periods in swine production, often leading to post-weaning growth lag and increased susceptibility to diseases [1]. The complex interplay of changes in this period includes psychological stress due to separation from the dam, environmental stresses from moving and mixing, dietary stress from a change in nutrient sources, and the developing immune system’s challenge to respond to pathogenic pressures [2]. A key consequence of these combined stresses is the disturbance of the intestinal microbiota, which plays an essential role in nutrient digestion, immune modulation, and protection against pathogens [3]. Therefore, interventions that influence the gut microbiome have become an area of intense research interest in the quest to improve weanling pig health and productivity.

Purslane (*Portulaca oleracea* L.) contains a diverse array of chemical components, including polysaccharides, organic acids, flavonoids, alkaloids, and terpenoids. This makes it a natural plant with a broad spectrum of pharmacological activities [4]. Pharmacological studies have demonstrated that purslane possesses a variety of therapeutic effects. Clinically, it is used for its anti-inflammatory, free-radical-scavenging, antibacterial, and immune-boosting properties [5,6]. In China, purslane is an important component of traditional Chinese medicine formulations due to its immunosuppressive and viral suppressive effects. For instance, purslane soup, which prominently features purslane, is primarily used to treat bacillary dysentery and enteritis. This therapeutic application is likely linked to the anti-inflammatory and immunomodulatory properties of purslane polysaccharides [4].

Fermentation is commonly utilized in the processing of traditional Chinese medicine [7,8], to enhance the therapeutic efficacy of these herbal remedies [9]. Fermentation can promote the conversion of the primary active components of medicinal herbs into their metabolites, potentially augmenting the therapeutic effects of herbal drugs [10,11]. Studies have shown that lactic acid bacteria fermentation could enrich the profile of biogenic compounds and enhance the functional features of common purslane [12]. In addition, a study also showed that probiotic-fermented purslane (FP) alleviated 2, 4-dinitrofluorobenzene (DNFB)-induced atopic dermatitis by inhibiting the NF-κB signaling pathway [13]. Thus, FP could potentially amplify these beneficial properties, making it a candidate for dietary supplementation in weaned piglets.

Considering the importance of gut health during weaning and the potential of fermented plant-based supplements, the present study investigated the effects of dietary supplementation with FP on growth performance, immune function, and fecal microbiota composition in weaned piglets. The hypothesis was that FP would promote growth performance and enhance immune responses by modulating the gut microbiome and increasing the production of functional microbial metabolites such as butyrate, known for its positive effects on gut health and immune function. This study aimed to provide a comprehensive assessment of how dietary FP affects the growth performance, health status, and microbial ecology of weaned piglets. The findings are expected to offer novel insights into the potential of fermented plant-derived dietary supplements to improve weanling pig management strategies, with implications for animal health and sustainable livestock production.

## 2. Materials and Methods

This study was carried out at Tianpeng Experimental Pig Farm, situated in Langfang, Hebei Province, China. The experimental protocols involving animals in this research were formally approved by the Institutional Animal Care and Use Committee (IACUC) at the Institute of Feed Research, Chinese Academy of Agricultural Sciences (approval No. IFR-CAAS20221003).

### 2.1. Fermented Purslane Preparation

*Bacillus subtilis* (ACCC 02136) was inoculated into a nutrient agar (NA) liquid medium and cultured at 30 °C for 24 h. *Sacccharomyces cerevisiae* (ACCC 21249) was inoculated into a yeast extract peptone glucose (YGP) liquid medium and incubated at 28 °C for 30 h. *Lactobacillus reuteri* (ACCC 03949) was seeded into a DeMan, Rogosa, and Sharpe (MRS) liquid medium and cultured at 37 °C for 30 h to prepare individual bacterial suspensions. 

Fresh purslane was air-dried and ground into powder. A 300 g sample was placed into a 1 L conical flask and sterilized at 121 °C under high pressure for 20 min. After cooling, 200 mL of *Bacillus subtilis* suspension (2.85 × 10^8^ CFU/mL), 200 mL of brewer’s yeast suspension (2.24 × 10^8^ CFU/mL), and 200 mL of *Lactobacillus reuteri* suspension (1 × 10^8^ CFU/mL) were sequentially added. The mixture was evenly stirred and left to ferment open at room temperature for 7 days and then was left to air-dry naturally for future use. The main nutritional composition and the level of short-chain fatty acids (SCFAs) of FP are shown in Table 1. The pH value of FP was measured by using a pH meter according to a previous study [14].

### 2.2. Experimental Animals and Diet

The study involved forty-eight Duroc × (Landrace × Large White) 28-day healthy weaned piglets with similar initial body weight (8.51 ± 0.75 kg). These piglets were randomly distributed into 16 pens, with three piglets per pen, all located within the same environmentally controlled room, which was maintained at a temperature of 26–28 °C. Half of the male and half of the female pigs in each group were included. The experimental design constituted two treatment groups, each comprising eight pens and three pigs in each pen. The control group (CON) was fed a standard corn–soybean meal basal diet, whereas the treatment group (FP) received the same basal diet supplemented with 0.20% FP through substitution with wheat bran. During the trial, all piglets had free access to fresh water and were allowed to feed ad libitum. The trial lasted 28 days. The experimental diets were formulated according to the National Research Council’s (NRC, 2012) nutritional recommendations. The detailed ingredients and the nutrition composition of the basal diets used in the study are outlined in Table 2.

### 2.3. Growth Performance, Diarrhea Incidence Measurement, and Sample Collection

All the experimental piglets were weighed individually early in the morning at the start of the trial (day 0) and subsequently on days 7, 14, and 28 to monitor their growth. Feed consumption for each pen was recorded throughout the entirety of the study. From these data, we calculated the average daily feed intake (ADFI), average daily gain (ADG), and feed-to-gain ratio (F/G). Diarrhea scores were recorded daily for all piglets from days 0 to 14 by the same individual, using a scale based on previously established methods [15]: 1 = well-formed feces, 2 = sloppy feces, and 3 = watery feces. Piglets with a score of 3 were considered to have diarrhea. The incidence of diarrhea (%) was calculated as the number of piglets with diarrhea divided by the total number of piglets in each group, expressed as a percentage. On days 14 and 28, one piglet from each pen, serving as one replicate, was randomly selected to sample blood from the anterior vena cava. The blood samples were collected in serum separator tubes, left to clot for at least 2 h at room temperature, and then centrifuged at 3000× *g* for 10 min to separate the serum. The obtained serum was then stored at −20 °C for subsequent assessments of immune function and antioxidant markers. At the end of the trial, one piglet from each pen was randomly selected for fresh feces collection. These samples were stored at −80 °C for subsequent microbial and SCFA analyses.

### 2.4. Analysis of Nutritional Composition in Fermented Purslane and Diet

The nutritional content of the FP and formulated diet samples was analyzed by adhering to the Official Methods of Analysis established by AOAC International (AOAC, 2006). The methodologies for detecting dry matter (DM), crude fat (EE), crude protein (CP), phosphorus (P), calcium (Ca), acid detergent fiber (ADF), neutral detergent fiber (NDF), and gross energy (GE) levels were conducted according to the detailed descriptions provided in our previously published article [16].

### 2.5. Detection of Immune and Antioxidant Indicators in Serum

The serum concentrations of immunoglobulins A (IgA), G (IgG), and M (IgM), as well as the cytokines interleukin (IL)-10, IL-6, tumor necrosis factor (TNF)-α, and IL-1β, were measured by using commercial ELISA kits (Shanghai Enzyme-linked Biotechnology Co., Ltd., Shanghai, China). Enzymatic activities of superoxide dismutase (SOD) and catalase (CAT), along with total antioxidant capacity (T-AOC) and malondialdehyde (MDA) levels in serum, were determined with commercial assay kits (Beijing Solarbio Science & Technology Co., Ltd., Beijing, China). D-lactate levels and diamine oxidase (DAO) activity in serum samples were evaluated by using commercial kits (Beijing Welab Biotechnology Co., Ltd., Beijing, China). All measurements followed the manufacturers’ protocols.

### 2.6. 16S rRNA Sequencing and Analysis of Fecal Microbiota

Genomic DNA from the total microbial community within the fecal samples was isolated by utilizing the QIAamp Fast DNA Stool Mini Kit (Qiagen, Hilden, Germany). The V3–V4 hypervariable regions of the 16S rRNA gene were selectively amplified by employing the primers 341F (5′-ACTCCTACGGGAGGCAGCAG-3′) and 806R (5′-GGACTACHVGGGTWTCTAAT-3′), following previously described protocols [17]. The resultant PCR products underwent a purification process, after which they were quantified and pooled in equimolar concentrations to prepare for PE300 sequencing conducted on the Illumina MiSeq platform (Illumina, San Diego, CA, USA), in line with the standardized methodologies of Majorbio Bio-Pharm Technology Co. Ltd. (Shanghai, China). The acquired raw sequencing data were processed via QIIME 2, implemented on the Majorbio I-Sanger Cloud Platform accessed on 14 February 2023 (https://www.i-sanger.com/). The sequences underwent quality control and were denoised by using DADA2 with the default parameters, producing amplicon sequence variants (ASVs) [18]. The ASVs were considered valid only if they had at least two reads and were present across multiple samples. The alpha and beta diversities within the samples were computed by using the vegan package (version 3.3.1). Principal Coordinates Analysis (PCoA) was performed by utilizing Bray–Curtis distances to elucidate the compositional dissimilarities among the microbial communities. Taxonomic differentiation among samples was identified through LEfSe (linear discriminant analysis effect size) and was corroborated by BLAST searches against the NCBI 16S rRNA sequence database. The functional potential of the intestinal microbiota was predicted by using PICRUSt2. All sequence reads from this study have been deposited in the NCBI Sequence Read Archive (SRA) under accession number SRP497541.

### 2.7. Determination of Fecal Short-Chain Fatty Acids

The quantification of SCFAs in FP and fecal samples was conducted by utilizing ion chromatography, following the procedures delineated in a recent study [19]. In brief, about 0.2 g of FP and fecal matter were solubilized in chilled dilution water, enriched with zinc sulfate heptahydrate (ZnSO_4_·7H_2_O) and potassium ferricyanide (K_4_Fe(CN)_6_·3H_2_O). This mixture was then agitated for 30 min and subsequently subjected to a centrifugation process at 10,000 rpm for 10 min at 4 °C. The post-centrifugation step involved filtration of the sample, followed by the dilution of the collected supernatant with distilled water in a 1:4 ratio. The final step entailed the analysis of the prepared supernatant for SCFA content by employing the 940 Professional IC Vario Ion Chromatograph (IC; Metrohm, Herisau, Switzerland).

### 2.8. Statistical Analysis

The statistical design of the experiment is completely randomized. The pen was used as the experimental unit for determining growth performance. For the analysis of serum immune and antioxidant parameters and fecal SCFA concentrations, the individual pig was recognized as the test unit. These assessments were performed by utilizing the IBM SPSS Statistics 20.0 software tool (SPSS Inc., Chicago, IL, USA). The differences in growth performance indicators, serum immune and antioxidant indicators, and fecal SCFA levels were identified through Student’s *t*-test. It was considered significant when *p* < 0.05, and there was a significant tendency when 0.05 ≤ *p* < 0.10. The Spearman correlation test was employed to evaluate the relationships among the fecal microbiota composition, microbial butyrate synthesis-related enzymes, and SCFA levels. For these analyses, Graphpad Prism version 5.0 (Graphpad Software, San Diego, CA, USA) served as the primary analytical software.

## 3. Results

### 3.1. Growth Performance and Diarrhea

**Figure 1:**
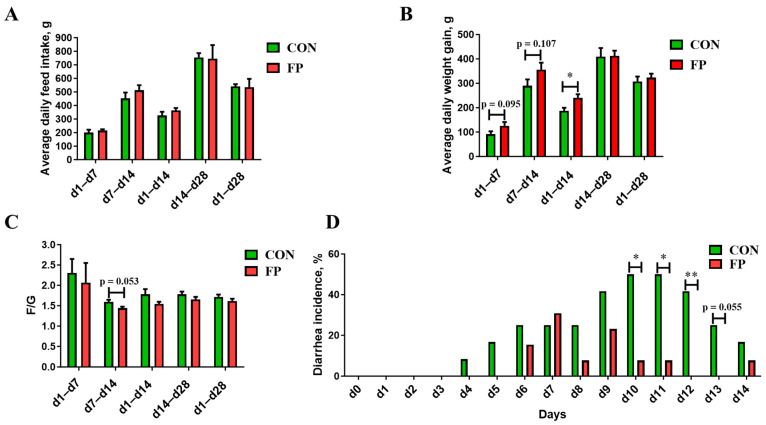
Effects of dietary supplementation of FP on growth performance and diarrhea rate in weaning piglets (*n* = 8). (**A**) Average daily feed intake; (**B**) average daily weight gain; (**C**) F/G; (**D**) diarrhea incidence. CON, piglets that were fed a standard corn–soybean meal basal diet; FP, piglets that were fed a basal diet supplemented with 0.20% fermented purslane. All data are presented as means ± SEM. *, *p* < 0.05; **, *p* < 0.01.

As shown in Figure 1, while the inclusion of FP in the diet did not influence the ADFI of weaned piglets when compared to the CON group, it significantly boosted the ADG over the first 14 days following weaning (*p* < 0.05). No impact on ADG was recorded from day 14 to day 28 (*p* > 0.05). A trend toward improved F/G was observed from days 7 to 14 of the trial period (0.05 ≤ *p* < 0.10). Additionally, FP supplementation markedly diminished the occurrence of diarrhea in the trial’s later stage, with a significant reduction noted specifically from days 10 to 13 (*p* < 0.05).

### 3.2. Immune Modulation and Antioxidant Effects

**Figure 2:**
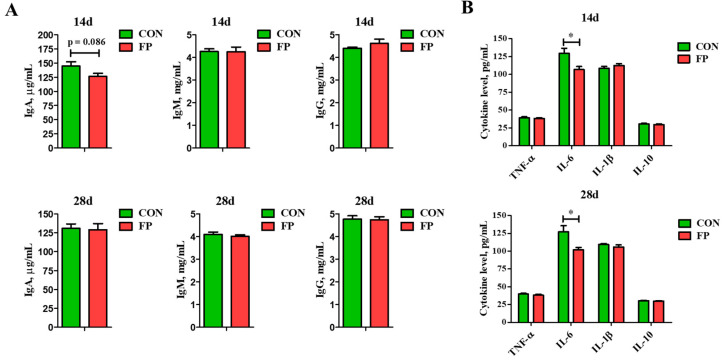
Effects of dietary supplementation of FP on immune indicators in weaning piglets (*n* = 8). (**A**) Serum immunoglobulin levels; (**B**) serum cytokines level. IgA, immunoglobulin A; IgG, immunoglobulin G; IgM, immunoglobulin M; TNF-α, tumor necrosis factor-α; IL-6, interleukin 6; IL-1β, interleukin 1β; IL-10, interleukin 10; CON, piglets that were fed a standard corn–soybean meal basal diet; FP, piglets that were fed a basal diet supplemented with 0.20% fermented purslane. All data are presented as means ± SEM. *, *p* < 0.05.

As illustrated in Figure 2, regarding immune modulation, FP showed no significant effect on the levels of IgA, IgG, and IgM in the serum on days 14 and 28 when compared to the control group (*p* > 0.05). However, there was a noted trend towards reduced IgA levels in the serum on day 14 (0.05 ≤ *p* < 0.10). FP significantly lowered the concentrations of IL-6 in the serum by both day 14 and day 28 post-weaning (*p* < 0.05). There were no considerable changes observed in the levels of serum IL-1β, TNF-α, and IL-10 on day 14, nor were there significant effects on serum TNF-α, IL-1β, and IL-10 levels on day 28. 

**Figure 3:**
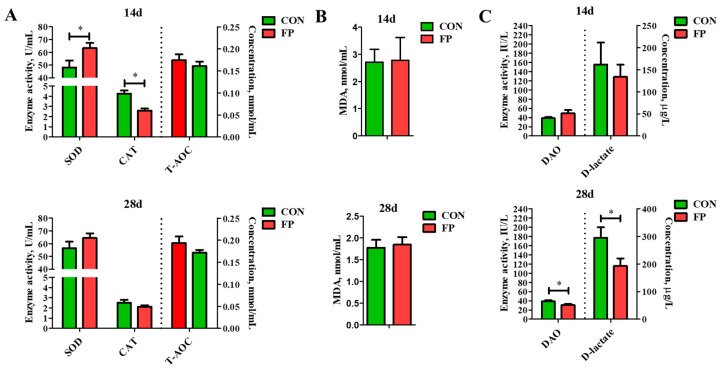
Effects of dietary supplementation of FP on antioxidant indicators and intestinal permeability indicators of weaning piglets (*n* = 8). (**A**) Activity of SOD and CAT and concentration of T-AOC in serum; (**B**) concentration of MDA in serum; (**C**) activity of DAO and concentration of D-lactate in serum. SOD, superoxide dismutase; CAT, catalase; T-AOC, total antioxidant capacity; MDA, malondialdehyde; DAO, diamine oxidase; CON, piglets that were fed a standard corn–soybean meal basal diet; FP, piglets that were fed a basal diet supplemented with 0.20% fermented purslane. All data are presented as means ± SEM. *, *p* < 0.05.

In terms of antioxidant capacity (Figure 3), compared with the control group, FP substantially increased SOD activity in the serum by day 14 while decreasing CAT enzyme activity (*p* < 0.05), with no significant impact on T-AOC and MDA contents (*p* > 0.05). On day 28 post-weaning, FP did not significantly affect SOD activity, CAT activity, T-AOC, or MDA content in the serum (*p* > 0.05).

DAO and D-lactate are markers that reflect intestinal integrity. Compared to the control group, feeding FP did not significantly influence DAO activity or D-lactate levels in the serum on day 14 (*p* > 0.05). However, there was a significant reduction in their levels on day 28 (*p* < 0.05).

### 3.3. Fecal Microbiota Composition and Function

**Figure 4:**
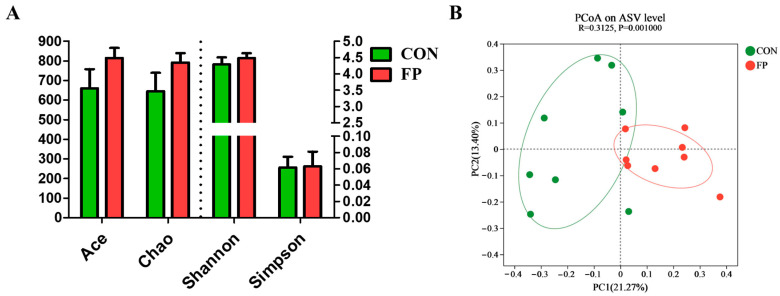
Effects of dietary supplementation of FP on fecal microbiota in weaning piglets (*n* = 8). (**A**) α-Diversity; (**B**) PCoA plot depicting β-diversity of fecal microbiota based on Bray–Curtis distance. CON, piglets that were fed a standard corn–soybean meal basal diet; FP, piglets that were fed a basal diet supplemented with 0.20% fermented purslane. All data are presented as means ± SEM.

**Figure 5:**
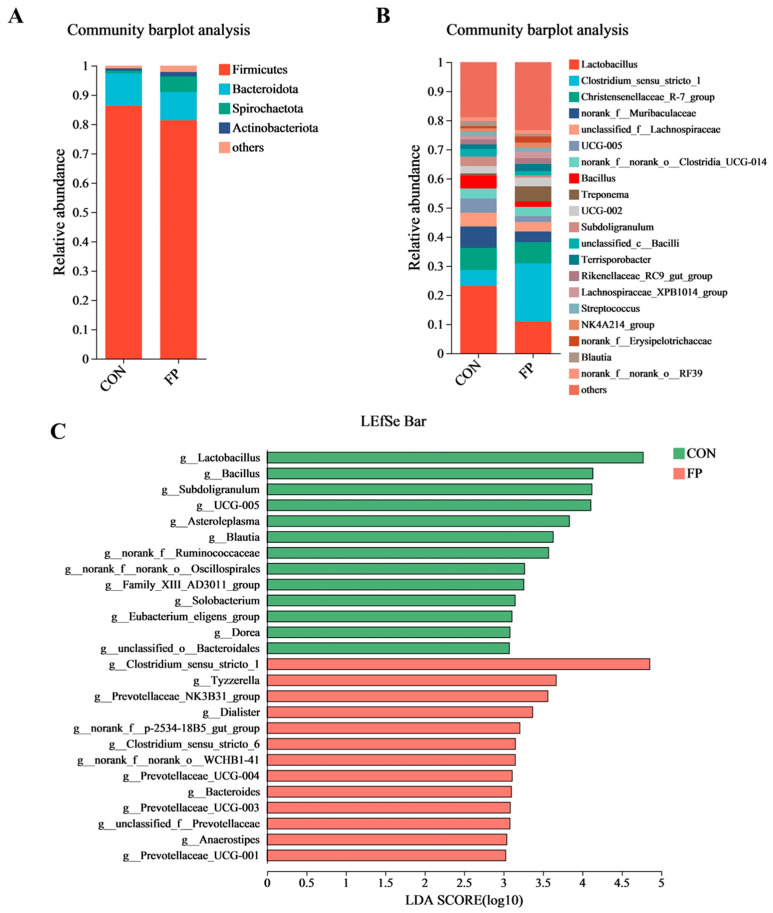
Effects of dietary supplementation of FP on fecal microbial composition in weaning piglets (*n* = 8). (**A**) Fecal microbiota composition shown at phylum level; (**B**) fecal microbiota composition shown at genus level; (**C**) LEfSe analysis of differential enrichment of fecal bacteria at genus level (linear discriminant analysis [LDA] > 3). CON, piglets that were fed a standard corn–soybean meal basal diet; FP, piglets that were fed a basal diet supplemented with 0.20% fermented purslane.

To evaluate the effects of diet supplementation with FP on the composition of gut microbiota in piglets, we performed 16S rRNA gene sequencing on fecal samples. Although FF administration did not significantly alter microbial α-diversity (*p* > 0.05) (Figure 4A), the PCoA based on Bray–Curtis distances revealed distinct clustering between the two piglet groups (ANOSIM, *p* < 0.05) (Figure 4B). The microbiota composition was predominantly characterized by two phyla, Firmicutes and Bacteroidota (Figure 5A), and was mainly composed of the genera *Lactobacillus*, *Clostridium_sensu*_stricto_1, and Christensenellaceae_R-7_group in both the CON and FP-treated groups (Figure 5B). At the genus level, the FF group exhibited a significant increase in the abundance of *Clostridium_sensu*_stricto_1, *Tyzzerella*, and Prevotellaceae_NK3B31_group and a decrease in *Lactobacillus*, *Bacillus*, and *Subdoligranulum* compared with the CON group (LDA score > 3) (Figure 5C).

**Figure 6:**
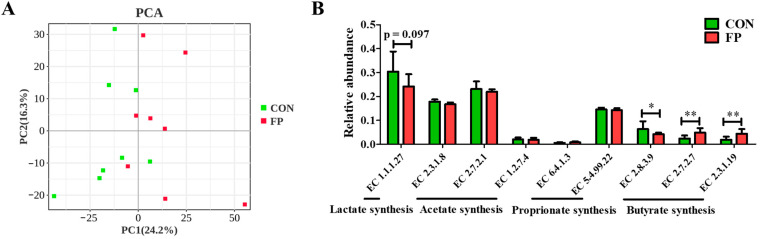
Effects of dietary supplementation of FP on fecal microbial enzymes based on the PICRUSt2 function prediction analysis in weaning piglets (*n* = 8). (**A**) The PCA plot is based on the enzymes; (**B**) the relative abundance of microbial enzymes related to SCFA synthesis. EC 1.1.1.27, L-lactate dehydrogenase; EC 2.3.1.8, phosphate acetyltransferase; EC 2.7.2.1, acetate kinase; EC 1.2.7.4, carbon-monoxide dehydrogenase (ferredoxin); EC 6.4.1.3, propionyl-CoA carboxylase; EC 5.4.99.22, methyl-malonyl-CoA mutase; EC 2.8.3.9, butyryl-CoA: acetate CoA-transferase; EC 2.7.2.7, butyrate kinase; EC 2.3.1.19, phosphate butyryl-transferase; CON, piglets that were fed a standard corn–soybean meal basal diet; FP, piglets that were fed a basal diet supplemented with 0.20% fermented purslane. All data are presented as means ± SD. *, *p* < 0.05; **, *p* < 0.01.

The functional predictions performed by PICRUSt2 suggested that FP had a considerable impact on the pattern of microbial enzymes, as evidenced by a PCA plot based on their relative abundance (Figure 6A). When we assayed enzymes involved in the synthesis of SCFAs (Figure 6B), we found no significant alterations in the relative abundance of enzymes associated with acetate (EC 2.3.1.8, EC 2.7.2.1, and EC 1.2.7.4) and propionate (EC 6.4.1.3 and EC 5.4.99.22) synthesis. However, the FP group revealed significant modulation in the butyrate production pathway, evidenced by increased relative abundance of EC 2.7.2.7 and EC 2.3.1.19 and decreased relative abundance of EC 2.8.3.9 (*p* < 0.05). There was also a trend indicative of modified lactate synthesis enzymes, demonstrated by a reduced relative abundance of EC 1.1.1.27 (*p* < 0.10).

### 3.4. Short-Chain Fatty Acid Levels

**Figure 7:**
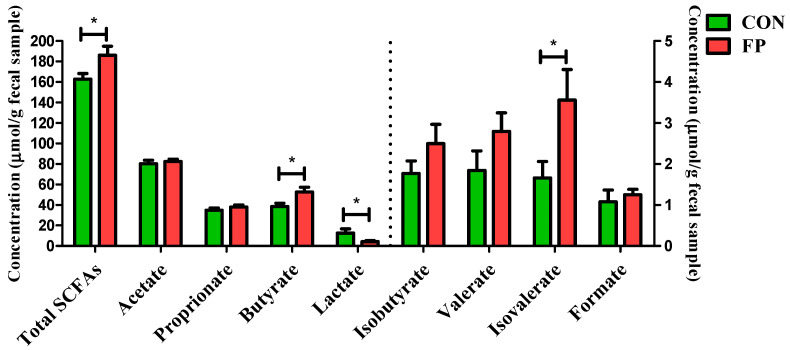
Effects of dietary supplementation of FP on concentration of SCFAs in feces of weaning piglets (*n* = 8). SCFAs, short-chain fatty acids; CON, piglets that were fed a standard corn–soybean meal basal diet; FP, piglets that were fed a basal diet supplemented with 0.20% fermented purslane. All data are presented as means ± SEM. *, *p* < 0.05.

To further investigate the changes in the microbial metabolism of SCFAs, we conducted a quantitative analysis of SCFA levels in feces. The dietary inclusion of FP markedly affected the profile of fecal SCFAs, as shown in Figure 7. Specifically, there was a marked increase in fecal total SCFAs, butyrate, and isovalerate concentrations and reduced fecal lactate concentration (*p* < 0.05), which are noteworthy for their roles in intestinal health and metabolism. Conversely, other SCFAs, such as acetate, propionate, isobutyrate, valerate, and formate, did not exhibit significant changes in response to the addition of FP to the diet (*p* > 0.05).

### 3.5. Correlation Analysis

**Figure 8:**
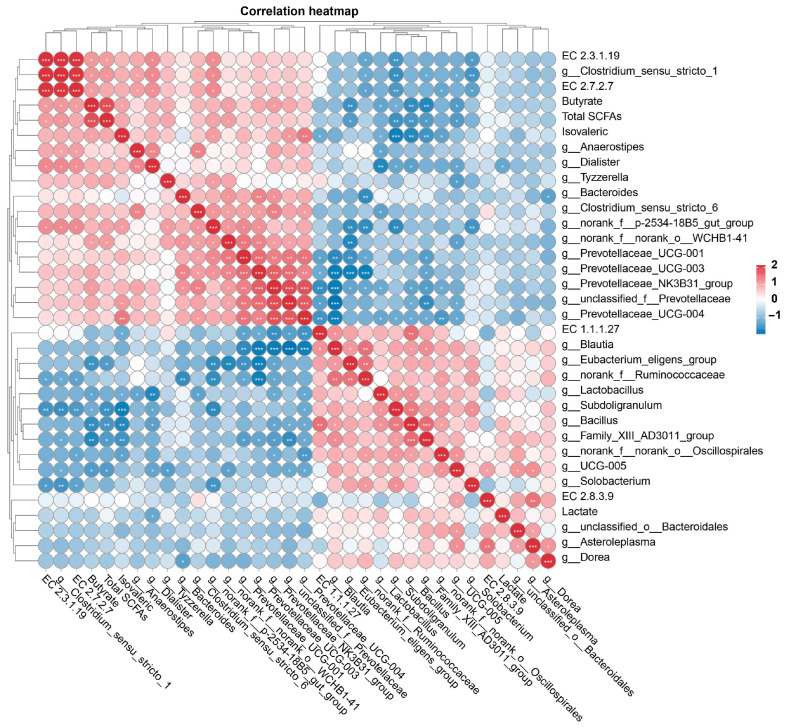
The Spearman correlations among the relative abundance of significantly altered fecal microbiota, microbiota encoding enzymes related to butyrate synthesis, and the concentration of SCFAs. EC 2.8.3.9, butyryl-CoA: acetate CoA-transferase; EC 2.7.2.7, butyrate kinase; EC 2.3.1.19, phosphate butyryl-transferase; SCFAs, short-chain fatty acids. *, *p* < 0.05; **, *p* < 0.01; ***, *p* < 0.001.

To investigate the relationships among microorganisms, SCFAs, and their respective metabolic enzymes, an additional Spearman correlation analysis was undertaken to better understand these interactions. As shown in Figure 8, the butyrate levels demonstrated a significant positive correlation with the relative abundance of EC 2.3.1.19, EC 2.7.2.7, and the *Clostridium_ sensu*_stricto_1 group. Inversely, they were negatively correlated with the relative abundance of EC 2.8.3.9, *Eubacterium_eligens*_group, *Lactobacillus*, *Subdoligranulum*, and *Bacillus* (*p* < 0.05). Additionally, the relative abundance of EC 2.3.1.19 was significantly positively correlated with *Clostridium_sensu*_stricto_1 and *Dialister*, while it was negatively correlated with *Subdoligranulum* and *Solobacterium* (*p* < 0.05). Furthermore, the relative abundance of EC 2.7.2.7 showed a significant positive correlation with *Clostridium_sensu*_stricto_1, *Anaerostipes*, and *Dialister* but a negative correlation with *Subdoligranulum* and *Solobacterium* (*p* < 0.05).

## 4. Discussion

The present study provides important insights into the potential beneficial effects of FP addition to the weanling diet of piglets to enhance the growth performance and intestinal health of weaned piglets. The observed improvement in ADG during the initial 14 days post-weaning supports the notion that FP may help mitigate the weaning stress that typically impairs growth in piglets [3]. This period is critical, as it marks the piglets’ transition to solid food and their adaptation to a new environment [20]. Although the change in F/G was not statistically significant, the trend towards better F/G from days 7 to 14 indicates improved feed efficiency, which can have cumulative benefits for overall weight gain and feed costs [21]. Though no significant change in feed intake was noted, this positive effect on ADG could be due to improved nutrient digestibility or gut health, as indicated by the subsequent results on immune modulation, antioxidant effects, and fecal microbiota composition.

The observed reduction in the incidence of diarrhea in the later stages of the trial could be linked to the overall improvement in gastrointestinal health, which is closely related to nutrient absorption efficiency. This observation aligns with prior studies suggesting that certain fermented foods can support intestinal health and reduce diarrheal instances in weaned piglets [22]. Notably, the significant reduction in the incidence of diarrhea from days 10 to 13 post-weaning is particularly noteworthy, as it points towards FP’s role in enhancing gut health, which may be attributed to the antimicrobial and modulatory properties of FP [23]. Diarrhea is commonly observed post-weaning due to factors like dietary changes and environmental stressors, leading to disruptions in the gut ecosystem and increased susceptibility to pathogens [24]. Therefore, FP’s ability to diminish diarrhea incidents highlights its potential to reduce the reliance on antibiotics—a critical issue in the context of growing antimicrobial resistance (AMR) concerns [25].

The immune system of weaned piglets is particularly susceptible to stressors, potentially leading to a challenged physiological state [26]. Our finding that FP did not significantly alter serum immunoglobulin levels (IgA, IgG, and IgM), combined with a reduction in IL-6, has implications for the anti-inflammatory response, which could be favorable for reducing inflammation during the transition period [27,28]. This is similar to findings that demonstrated the anti-inflammatory properties of FP [13,29]. The trend towards decreased IL-1β levels could also suggest the potential for fermented products to modulate the immune system subtly [30], although more investigation is needed to elucidate these relationships fully.

In terms of oxidative stress, the significant increase in SOD activity and the decrease in CAT enzyme activity on day 14 indicate that FP might alter antioxidant enzyme activities post-weaning, suggesting a potential benefit in managing oxidative stress [31]. SOD is an antioxidant enzyme responsible for catalyzing the conversion of superoxide anions (a harmful reactive oxygen species, ROS) into oxygen and hydrogen peroxide [32]. The increase in SOD activity suggests that FP may enhance the antioxidative defense mechanism against superoxide anions. This could indicate a beneficial impact of FP in improving the oxidative stress response in weaned piglets. CAT is another crucial antioxidant enzyme that breaks down hydrogen peroxide produced by SOD into water and oxygen [32]. The decrease in CAT enzyme activity in the present study may reflect an adaptive response to a change in diet composition. Since FP might introduce different types and amounts of bioactive compounds [4], piglets’ metabolic pathways might adjust accordingly. The body could be utilizing other antioxidant defenses more efficiently, or the CAT pathway might be downregulated in response to the specific types of ROS generated after FP consumption. Additionally, the changes observed could signify a phase of stress or adaptation to the introduced diet. The initial increase in ROS following the introduction of FP might trigger an enhanced SOD response to detoxify superoxide anions more effectively, while the decrease in CAT activity could suggest a temporary imbalance in handling hydrogen peroxide. Taken together, the significant increase in SOD activity and decrease in CAT enzyme activity in weaned piglets after feeding on FP can be interpreted as an adaptive response to the new diet, potentially indicating a shift in antioxidant defense strategies. The findings here are consistent with previous studies, which also reported alterations in antioxidant enzyme activities following the intake of FP [12,29]. These findings underscore the potential multifactorial benefits of FP, encompassing improvements in immune status and oxidative stress. The selective influence on these enzyme activities may underscore the complex interplay between dietary components and the piglets’ intrinsic antioxidant systems, which warrants further investigation.

Regarding the fecal microbiota, FP’s impact on the relative abundance of specific microbial populations points to a considerable alteration in the gut ecosystem. Although there is no direct evidence of the effect of FP on the gut microbiota of weaned piglets, its potential mechanisms for regulating gut microbiota include the following: Firstly, studies have reported that the components of purslane itself, for example, purslane polysaccharides, can regulate gut microbiota in rats [33]. Secondly, FP contains microbial fermentation metabolites such as SCFAs, which are competitively utilized by intestinal microorganisms, thereby controlling the microbial community structure [34]. The exact mechanism by which FP regulates the gut microbiota of weaned piglets still requires further investigation. In addition, the increase in *Clostridium_sensu*_stricto_1 and *Tyzzerella*, alongside the decrease in *Lactobacillus*, raises questions regarding the specific roles of these genera in gut health and warrants further investigation. Moreover, our results indicate that FP promotes distinct alterations in the composition of the gut microbiota, supporting the notion that fermented foods can shape the microbial community in beneficial ways [30,35], despite the lack of direct evidence regarding the impact of FP on gut microbiota regulation. The noted enhancement in the abundance of the *Clostridium_sensu*_stricto_1 group, a butyrate-producing bacterium known for its anti-inflammatory and health-promoting effects [36,37], coincided with decreased occurrence of diarrhea and bolstered immune responses in our piglets fed FP.

Phosphate butyryltransferase (EC 2.3.1.19) and butyrate kinase (EC 2.7.2.7) play a crucial role in the butyrate fermentation pathway, especially in converting various substrates, including carbohydrates and amino acids, into butyrate. Bacteria such as those in the *Clostridium* and *Fusobacterium* genera encode these enzymes. Our study observed a significant increase in the relative abundance of *Clostridium_sensu*_stricto_1 and bacteria associated with EC 2.3.1.19 and EC 2.7.2.7 in the FP group. Further correlation analysis revealed a positive relationship among *Clostridium_sensu*_stricto_1, EC 2.3.1.19, EC 2.7.2.7, and butyrate levels, suggesting that FP dietary supplementation could boost intestinal butyrate production through the proliferation of *Clostridium_sensu*_stricto_1. Consistently with previous research [36,38,39], we found a limited diversity of butyrate producers, identifying *Clostridium_sensu*_stricto_1 as key butyrate-forming bacteria in the gut. Butyrate, an SCFA, serves as a vital energy source for colonocytes, possesses anti-inflammatory properties, contributes to gut health, and may mitigate intestinal damage due to weaning stress in piglets [40]. Notably, our study revealed a significant reduction in serum markers of intestinal integrity (DAO and D-lactate levels) by day 28, indicating that FP has a positive, long-term effect on gut barrier function [41]. Maintaining a healthy intestinal barrier is essential to preventing pathogen translocation and systemic immune response [42]. The augmented butyrate production, potentially driven by the altered microbial activity due to FP supplementation, implies that FP may have prebiotic-like properties [43].

## 5. Conclusions

This study highlights that dietary supplementation with fermented purslane (FP) enhances early-growth performance by increasing weight gain and reducing diarrhea incidence in weaned piglets without affecting feed intake. Additionally, FP demonstrates potential immune-modulatory and antioxidant effects and specifically alters gut microbiota in favor of a shift in microbial metabolic activity toward butyrate production. These findings contribute valuable insights into the potential applicability of FP in weaned piglets’ diets as a strategy to improve early growth and health. Further research on the long-term effects and optimal dosages of FP could enhance its application in piglet diets.

## Figures and Tables

**Table 1:** Chemical components of fermented purslane (FP) (air-drying basis).

Item	FP
Nutrient level, %	
DM	85.23
CP	15.82
EE	0.09
NDF	49.73
ADF	31.22
Ca	0.75
Total P	0.55
GE, MJ/kg	10.17
SCFAs, mg/g	
Formate	0.13
Acetate	6.74
Propionate	0.11
Butyrate	ND
Isovaleric acid	0.16
Total SCFAs	9.35
Lactate, mg/g	2.21
pH value	9.33

Notes: DM = dry matter; CP = crude protein; EE = ether extract; NDF = neutral detergent fiber; ADF = acid detergent fiber; Ca = calcium; P = phosphorus; GE = gross energy; SCFAs = short-chain fatty acids; ND = not detected.

**Table 2:** Ingredient composition and nutrition components of the diets (%, as-fed basis).

Items	Experimental Phases and Treatments ^3^			
	d 0–14		d 14–28	
	CON	FP	CON	FP
Ingredients				
Corn	16.45	16.45	21.17	21.17
Extruded corn	32.00	32.00	40.00	40.00
Soybean meal (46% CP)	14.00	14.00	17.50	17.50
Extruded soybean	11.50	11.50	6.00	6.00
Fish meal	5.60	5.60	3.00	3.00
Whey powder	15.00	15.00	5.00	5.00
Soybean oil	1.00	1.00	1.20	1.20
Monocalcium phosphate	0.40	0.40	0.60	0.60
Limestone (CaCO_3_)	0.75	0.75	0.90	0.90
Salt	0.30	0.30	0.30	0.30
Choline chloride (60%)	0.05	0.05	0.05	0.05
L-Lysine HCl	1.20	1.20	1.08	1.08
DL-Methionine	0.09	0.09	0.08	0.08
Threonine	0.27	0.27	0.24	0.24
Tryptophan	0.02	0.02	0.01	0.01
Phytase	0.02	0.02	0.02	0.02
Acidifier	0.35	0.35	0.35	0.35
Zinc oxide	0.20	0.20	-	-
Antioxidant	0.02	0.02	0.02	0.02
Vitamin and mineral premix ^1^	0.05	0.05	0.05	0.05
Mineral premix ^1^	0.20	0.20	0.20	0.20
Wheat bran	0.53	0.33	2.23	2.03
Fermented purslane	-	0.20	-	0.20
Total	100.00	100.00	100.00	100.00
Analyzed nutrient level, %				
Dry matter	91.83	91.74	90.19	90.27
Crude protein	19.11	19.57	17.93	18.14
Calcium	0.89	0.86	0.97	0.95
Phosphorus	0.53	0.55	0.57	0.58
Calculated nutrient level, %				
ME, MJ/kg	14.23	14.23	14.02	14.02
SID Lysine ^2^	1.30	1.30	1.15	1.15
SID Methionine	0.38	0.38	0.34	0.34
SID Threonine	0.76	0.76	0.68	0.68
SID Tryptophan	0.21	0.21	0.19	0.19

Notes: ^1^ The vitamin and mineral premix supplied per kg of diet: niacin, 38.4 mg; calcium pantothenate, 25 mg; folic acid, 1.68 mg; biotin, 0.16 mg; vitamin A, 35.2 mg; vitamin B1, 4 mg; vitamin B2, 12 mg; vitamin B6, 8.32 mg; vitamin B12, 4.8 mg; vitamin D3, 7.68 mg; vitamin E, 128 mg; vitamin K3, 8.16 mg; zinc (ZnSO_4_·H_2_O), 110 mg; copper (CuSO_4_·5H_2_O), 125 mg; selenium (Na_2_SeO_3_), 0.19 mg; iron (FeSO_4_·H_2_O), 171 mg; cobalt (CoCl_2_), 0.19 mg; manganese (MnSO_4_·H_2_O), 42.31 mg; iodine (Ca(IO_3_)_2_), 0.54 mg. ^2^ SID = standardized ileal digestibility. ^3^ CON, piglets that were fed a standard corn–soybean meal basal diet; FP, piglets that were fed a basal diet supplemented with 0.20% fermented purslane.

## Data Availability

All sequence reads of fecal microbiota have been deposited in the NCBI Sequence Read Archive (SRA) under accession number SRP497541. Other data supporting the research findings can be requested from the corresponding author.
